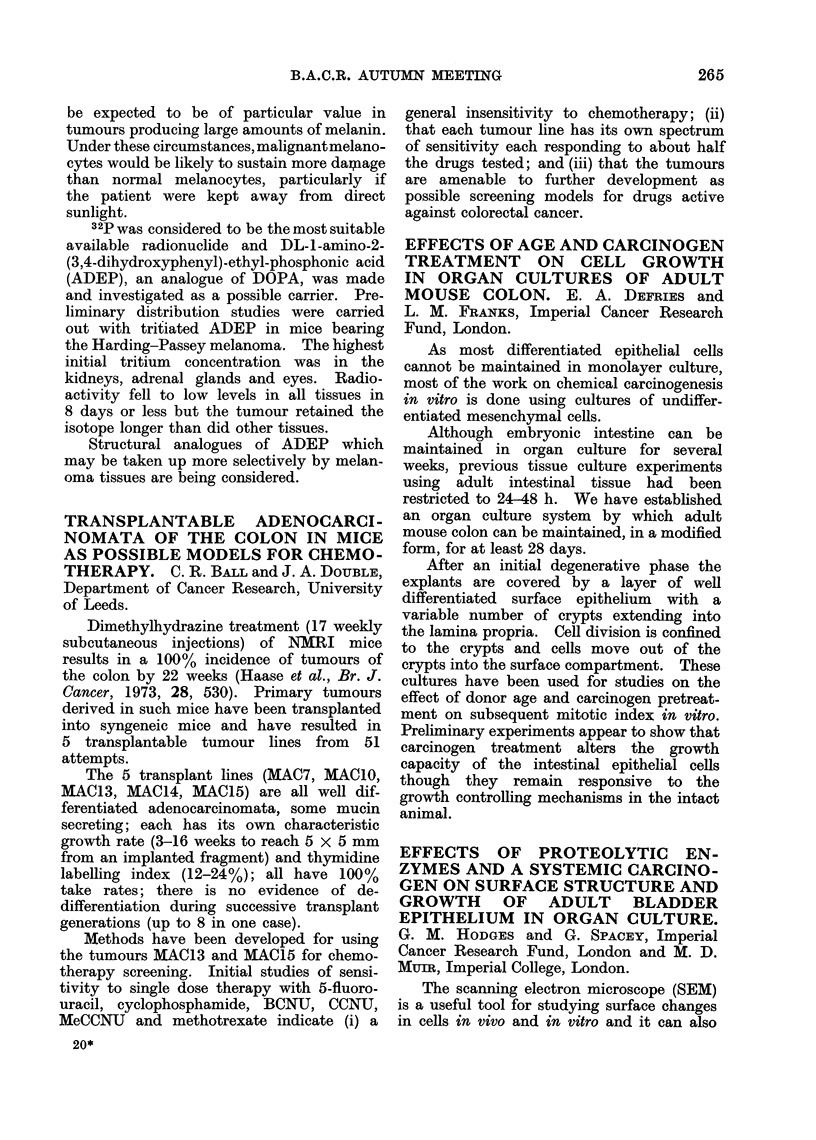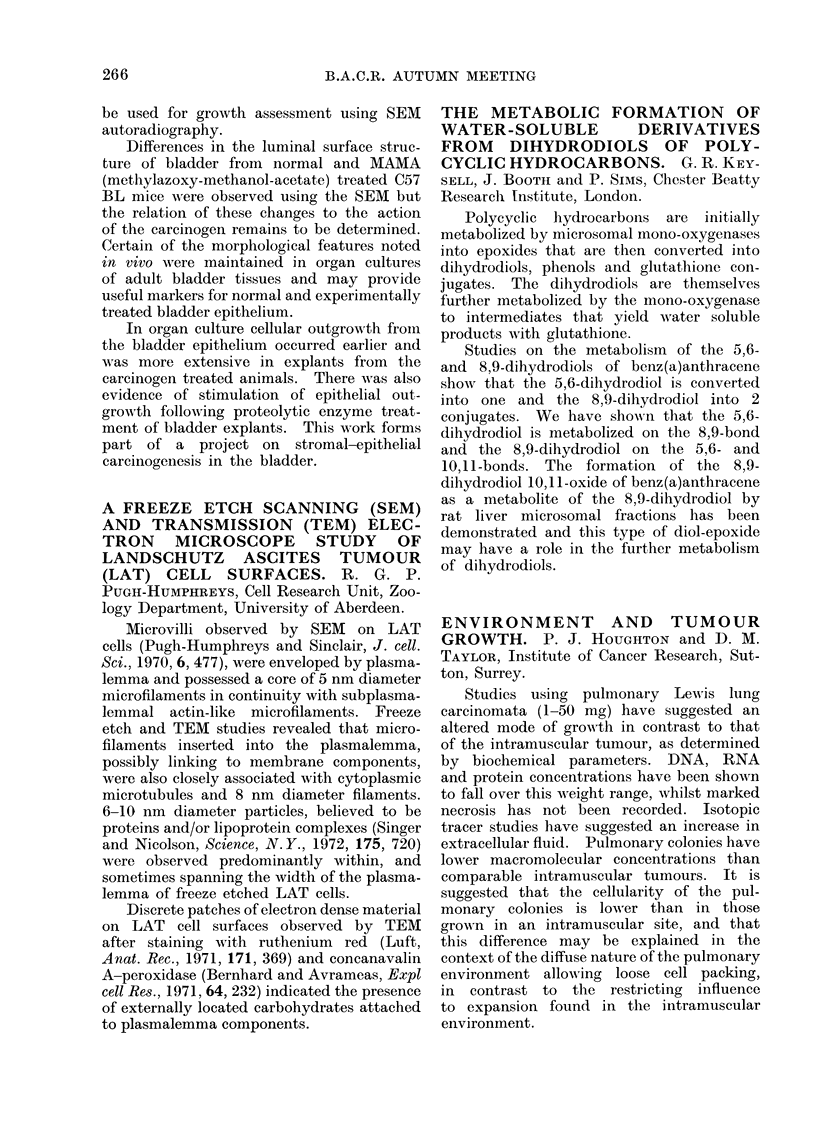# Effects of proteolytic enzymes and a systemic carcinogen on surface structure and growth in organ cultures of adult mouse colon.

**DOI:** 10.1038/bjc.1975.55

**Published:** 1975-02

**Authors:** G. M. Hodges, G. Spacey


### EFFECTS OF PROTEOLYTIC ENZYMES AND A SYSTEMIC CARCINOGEN ON SURFACE STRUCTURE AND GROWTH OF ADULT BLADDER EPITHELIUM IN ORGAN CULTURE.

G. M. Hodges and G. Spacey, Imperial Cancer Research Fund, London and M. D. Muir, Imperial College, London.

The scanning electron microscope (SEM) is a useful tool for studying surface changes in cells *in vivo* and *in vitro* and it can also be used for growth assessment using SEM autoradiography.

Differences in the luminal surface structure of bladder from normal and MAMA (methylazoxy-methanol-acetate) treated C57 BL mice were observed using the SEM but the relation of these changes to the action of the carcinogen remains to be determined. Certain of the morphological features noted *in vivo* were maintained in organ cultures of adult bladder tissues and may provide useful markers for normal and experimentally treated bladder epithelium.

In organ culture cellular outgrowth from the bladder epithelium occurred earlier and was more extensive in explants from the carcinogen treated animals. There was also evidence of stimulation of epithelial outgrowth following proteolytic enzyme treatment of bladder explants. This work forms part of a project on stromal–epithelial carcinogenesis in the bladder.